# A pigtailed macaque model of Kyasanur Forest disease virus and Alkhurma hemorrhagic disease virus pathogenesis

**DOI:** 10.1371/journal.ppat.1009678

**Published:** 2021-12-02

**Authors:** Rebecca M. Broeckel, Friederike Feldmann, Kristin L. McNally, Abhilash I. Chiramel, Gail L. Sturdevant, Jacqueline M. Leung, Patrick W. Hanley, Jamie Lovaglio, Rebecca Rosenke, Dana P. Scott, Greg Saturday, Fadila Bouamr, Angela L. Rasmussen, Shelly J. Robertson, Sonja M. Best

**Affiliations:** 1 Laboratory of Virology, Rocky Mountain Laboratories, National Institute of Allergy and Infectious Diseases, National Institutes of Health, Hamilton, Montana, United States of America; 2 Rocky Mountain Veterinary Branch, National Institute of Allergy and Infectious Diseases, National Institutes of Health, Hamilton, Montana, United States of America; 3 Research Technologies Branch, National Institute of Allergy and Infectious Diseases, National Institutes of Health, Hamilton, Montana, United States of America; 4 Laboratory of Molecular Microbiology, National Institute of Allergy and Infectious Diseases, National Institutes of Health, Bethesda, Maryland, United States of America; 5 Vaccine and Infectious Disease Organization, University of Saskatchewan, Saskatoon, Saskatchewan, Canada; 6 Center for Global Health Science and Security, Georgetown University, Washington, District of Columbia, United States of America; La Jolla Institute for Allergy and Immunology, UNITED STATES

## Abstract

Kyasanur Forest disease virus (KFDV) and the closely related Alkhurma hemorrhagic disease virus (AHFV) are emerging flaviviruses that cause severe viral hemorrhagic fevers in humans. Increasing geographical expansion and case numbers, particularly of KFDV in southwest India, class these viruses as a public health threat. Viral pathogenesis is not well understood and additional vaccines and antivirals are needed to effectively counter the impact of these viruses. However, current animal models of KFDV pathogenesis do not accurately reproduce viral tissue tropism or clinical outcomes observed in humans. Here, we show that pigtailed macaques (*Macaca nemestrina*) infected with KFDV or AHFV develop viremia that peaks 2 to 4 days following inoculation. Over the course of infection, animals developed lymphocytopenia, thrombocytopenia, and elevated liver enzymes. Infected animals exhibited hallmark signs of human disease characterized by a flushed appearance, piloerection, dehydration, loss of appetite, weakness, and hemorrhagic signs including epistaxis. Virus was commonly present in the gastrointestinal tract, consistent with human disease caused by KFDV and AHFV where gastrointestinal symptoms (hemorrhage, vomiting, diarrhea) are common. Importantly, RNAseq of whole blood revealed that KFDV downregulated gene expression of key clotting factors that was not observed during AHFV infection, consistent with increased severity of KFDV disease observed in this model. This work characterizes a nonhuman primate model for KFDV and AHFV that closely resembles human disease for further utilization in understanding host immunity and development of antiviral countermeasures.

## Author summary

Kyasanur Forest disease virus (KFDV) and Alkhurma hemorrhagic disease virus (AHFV) are tick-borne flaviviruses that cause viral hemorrhagic fevers in India and the Arabian Peninsula, respectively. Bonnet macaques and black-faced langurs are susceptible to KFDV infection, but these animals do not experience hemorrhagic signs as seen in human cases with KFDV. This work characterizes for the first time experimental infection of KFDV and AHFV in pigtailed macaques (PTMs). Infected PTMs can develop moderate to severe disease that models multiple features of human disease, including some hemorrhagic signs. Together these data describe the PTM model for KFDV and AHFV as a valuable tool for future work to study viral pathogenesis and for assessing the efficacy of vaccines and antivirals.

## Introduction

Tick-borne flaviviruses (TBFVs) can cause encephalitis or hemorrhagic fevers in humans and are considered emerging in parts of Asia, India, Europe and North America. Transmission of these viruses to humans primarily occurs following the bite of infected ticks. Specific viruses of concern include tick-borne encephalitis virus (TBEV), Powassan virus (POWV), Kyasanur Forest disease virus (KFDV), Alkhurma hemorrhagic fever virus (AHFV) and Omsk hemorrhagic fever virus (OHFV). In particular, KFDV and AHFV pathogenesis is not well understood. AHFV and KFDV cause fatal hemorrhagic fevers in humans, are listed as NIAID category C priority pathogens, and require maximum containment facilities to conduct research. KFDV was originally discovered in the Shimoga district of Karnataka state in India, but has expanded its geographical distribution in the last decade to include Kerala, Goa, and Maharashtra states [1]. An estimated 100–900 cases of KFDV occur annually with a 2–10% case-fatality rate [1–3]. KFDV causes a sudden onset of acute febrile illness including headache, fever, vomiting, diarrhea, inflammation of the eyes, and dehydration. Disease may progress to include hemorrhagic signs particularly bleeding from nose, gums, gastrointestinal tract, or lungs [3]. AHFV is a newly emerging genetic variant of KFDV that was first isolated in 1995 [4, 5]. While KFDV is localized to southwest India, AHFV cases have been identified in Egypt and Saudi Arabia [4, 6]. AHFV causes similar febrile illness and hemorrhagic disease in humans as observed with KFDV [7]. However, very little is known regarding mechanisms of disease including how the virulence potential compares between the two virus species.

The expanding geographic range of TBFVs witnessed over the last several decades, the steady increase in annual cases, and the potential for severe clinical disease necessitates the development of relevant animal models to study viral pathogenesis and to evaluate vaccines and antiviral therapies [1, 8, 9]. In the case of KFDV and AHFV, small animal models have limitations in that they do not recapitulate key aspects of human disease. Several commonly available strains of laboratory mice are susceptible to KFDV and AHFV infection, with mice developing neurological signs 5–10 days after infection associated with high virus burden in the brain at clinical endpoints [10–14]. However, the neurotropic nature of KFDV and AHFV in the mouse model does not recapitulate the hemorrhagic nature of human disease. Other commonly used small animal models (ferrets, guinea pigs, and hamsters) do not show signs of disease following infection with KFDV, despite the presence of virus in the brain of infected hamsters [15].

Non-human primate (NHP) models have not been described for AHFV, while several NHP species have been inoculated with KFDV, with susceptibility to infection being highly species-dependent. Early reports suggested that rhesus macaques (*Macaca mulatta)* infected with KFDV may develop viremia but clinical signs of disease were absent [3, 16]. Red-faced bonnet macaques (*Macaca radiata*) and black-faced langurs (*Presbytis entellus*) are naturally susceptible to KFDV, and clusters of KFDV human cases coincide with discovery of deceased bonnet macaques and langurs in local forested areas [3]. Experimental infection of these two NHP species demonstrated that black-faced langurs in particular are highly susceptible to KFDV and succumbed to infection during the viremic phase [17]. Bonnet macaques infected with KFDV experience a biphasic illness with 10–100% lethality depending on the study [17–20]. The acute phase is characterized by dehydration, hypotension, bradycardia and diarrhea, while neurological signs such as tremors may be observed in the second phase [17, 18, 20, 21]. However, no hemorrhagic signs were reported in these animals associated with mild decreases in platelets [18, 19]. In addition, infected bonnet macaques develop a second neurological disease phase more frequently that human KFDV disease, with KFDV routinely isolated from macaque brain and CSF [18, 21]. Finally, while bonnet macaques and black-faced langurs have application to studying KFDV disease, these species are not readily available for biomedical research outside of India, thus preventing their wider use as a comparative model for KFDV.

The mild nature of KFDV infection in rhesus macaques is likely to be multifaceted. However, rhesus macaques possess a restriction factor called tripartite motif protein 5 (TRIM5) that is well known for its ability to prevent HIV replication through its recognition of the retroviral capsid lattice [22, 23]. Recently, our group showed that TRIM5 is also strongly restrictive to TBFVs, including KFDV, through TRIM5 interaction with the viral protease and RNA helicase NS2B/3 [24]. Unlike rhesus macaques, the TRIM5 gene of pigtailed macaques (PTMs) has undergone a retrotransposition event where cyclophilin A (CypA) replaces the B30.2/PRY/SPRY domain of TRIM5 primarily responsible for interactions with viral substrates [25]. This renders PTM TRIM-Cyp non-restrictive to retroviruses [26], and most likely also prevents recognition of the flavivirus NS2B/3 protein [24]. The lack of TRIM5-mediated restriction in PTMs was important for the development of a NHP model for HIV-1[26]. Hence, our ongoing hypothesis is that the PTM TRIM-Cyp fusion protein is non-restrictive to TBFVs and that this may reduce intrinsic barriers to infection and predispose PTMs to clinical disease following inoculation with KFDV and AHFV.

In an effort to develop improved disease models, we investigated whether PTMs were susceptible to infection with KFDV and AHFV. KFDV- and AHFV-infected PTMs displayed moderate to severe clinical illness with hemorrhagic signs. The PTM KFDV and AHFV models closely follow reports of KFDV and AHFV infection in humans and capture aspects of human disease that have not been described in other KFDV NHP models. Transcriptomic analysis in blood of PTMs inoculated with KFDV or AHFV revealed differences in clotting factor downregulation that may contribute to enhanced pathogenesis following inoculation with KFDV compared to AHFV. Thus PTMs represent an important model for countermeasure development against these emerging viruses.

## Results

### PTM TRIM-Cyp is not a replication barrier for KFDV

KFDV is sensitive to restriction by rhesus TRIM5 dependent on the B30.2/PRY/SPRY domain, but it is unknown whether PTM TRIM-Cyp can restrict replication. PTM TRIM-Cyp was cloned into a lentiviral expression vector with an HA tag and used to generate a stable HEK293 cell line (S1A Fig). These cells were infected with KFDV, and virus in supernatants was collected and titered on Vero cells by plaque assay (S1B Fig). While KFDV growth was restricted in cells expressing rhesus TRIM5, the PTM TRIMCyp did not impact release of infectious virus, demonstrating that PTM TRIM-Cyp is not a restriction factor for KFDV, consistent with previous data showing TRIM-Cyp fusion proteins are not functional against TBFVs [24].

### Pilot studies of KFDV infection of PTMs

To determine whether PTMs are susceptible to infection with KFDV, a pilot experiment was performed where two animals were inoculated subcutaneously (sc) to mimic a tick bite between the shoulder blades with 10^5^ pfu KFDV P9605. The absolute inoculum delivered through the bite of hard ticks is not known due to the extended duration of feeding, and hence this virus dose was selected based on the range of 10^4^−10^6^ pfu demonstrated for flaviviruses inoculated by mosquito bite [27, 28]. Animals were scored for clinical signs of disease by trained veterinary staff based on a number of categories including appetite, general appearance, respiration, and activity level. Higher scores indicate more severe disease, and a score of 35 or greater is the pre-established criteria for euthanasia. Both KFDV-infected animals (KFDV 1 and KFDV 2) showed clinical signs (S1 Table), although these were not consistent between the two KFDV-infected animals (S2 Fig). KFDV 1 had decreased appetite, piloerection, and hunched posture whereas KFDV 2 had decreased appetite and transient bloody nasal discharge. Animals had fully recovered by 14 dpi, and they did not develop any additional clinical signs through day 43. Viral RNA was detected in the plasma at 2, 4, and 6 dpi by qRT-PCR. Infectious virus was not detected in the plasma of KFDV 1, but virus was directly isolated from the plasma of KFDV 2 at the peak of viremia at 4 dpi. Neutralizing antibodies were detected in KFDV-infected animals starting at 8–10 dpi. Immune sera from KFDV 1 cross-neutralized AHFV by a focus reduction neutralization test (S2 Fig), consistent with the cross-neutralization of related flavivirus serogroups in tissue culture and *in vivo* [11, 29, 30]. No significant lesions were noted at necropsy, except KFDV 1 had a pale, reticulated liver (Table 1). This pilot study demonstrated that PTMs are susceptible to KFDV infection and have the potential to develop viremia, but suggested that an sc inoculation may result in highly variable clinical outcomes.

**Table 1:** KFDV and AHFV Necropsy findings.

	KFDV 1 (sc)	KFDV 2 (sc)	KFDV 3 (sc/iv)	KFDV 4 (sc/iv)	KFDV 5 (sc/iv)	KFDV 6 (sc/iv)	KFDV 7 (sc/iv)	KFDV 8 (sc/iv)	AHFV 1 (sc/iv)	AHFV 2 (sc/iv)	AHFV 3 (sc/iv)	AHFV 4 (sc/iv)
**Day post infection**	D42	D42	D6	D8	D8	D9	D9	D7	D9	D9	D9	D8
**Discharge**	Normal	Normal	Normal	Epistaxis	Epistaxis	Dried blood in nasal cavity	None	Epistaxis	None	Dried blood in nasal cavity	Dried blood in nasal cavity	None
**Subcutis**	Normal	Normal	Normal	Tacky	Normal	Tacky	Normal	Tacky	Tacky	Normal	Tacky	Edema, Tacky
**Liver**	Pale	Normal	Yellow-orange	Pale	Pale	Pale	Pale tan	Pale	Pale	Normal	Normal	Pale
	Reticulated		Fatty	Friable	Reticulated	Enlarged	Enlarged	Reticulated	Enlarged			Enlarged
			Reticulated	Fatty	Fatty	Rounded Edges	Swollen	Prominent Vasculature	Rounded Edges			Lumpy
									Multifocal Hemorrhages			
**Spleen**	Normal	Normal	Adhesions to mesenteric	Enlarged, firm	Normal	Normal	Normal	Normal	Lumpy Irregular surface	Normal	Enlarged	Enlarged, Turgid
**Lungs**	Normal	Normal	All lobes hyperemic	Congested	Normal	Multifocal <1mm black spots	Adhesions	Normal	Hemorrhagic, dorsal ecchymosis all lobes, caudal lobes ventral surface	Failure to collapse	Normal	Small bulla on R lower lung lobe
				Left lung all lobes hyperemic								
**Adhesions**	Normal	Normal	Adhesions throughout abdomen	Mesenteric adhesions	Normal	Normal	Adhesions in chest and lung cavity	Multiple Adhesions lower abdomen	Normal	Normal	Normal	Normal
**Other Comments**				Jejunum fluid-filled	Large parotid salivary glands			Mediastinal LN edematous			Dehydrated	Dehydrated
				Foam in trachea				Gall bladder distended				

### Clinical signs and tissue distribution of virus in KFDV-infected PTMs following sc/iv inoculation

**Fig 1:**
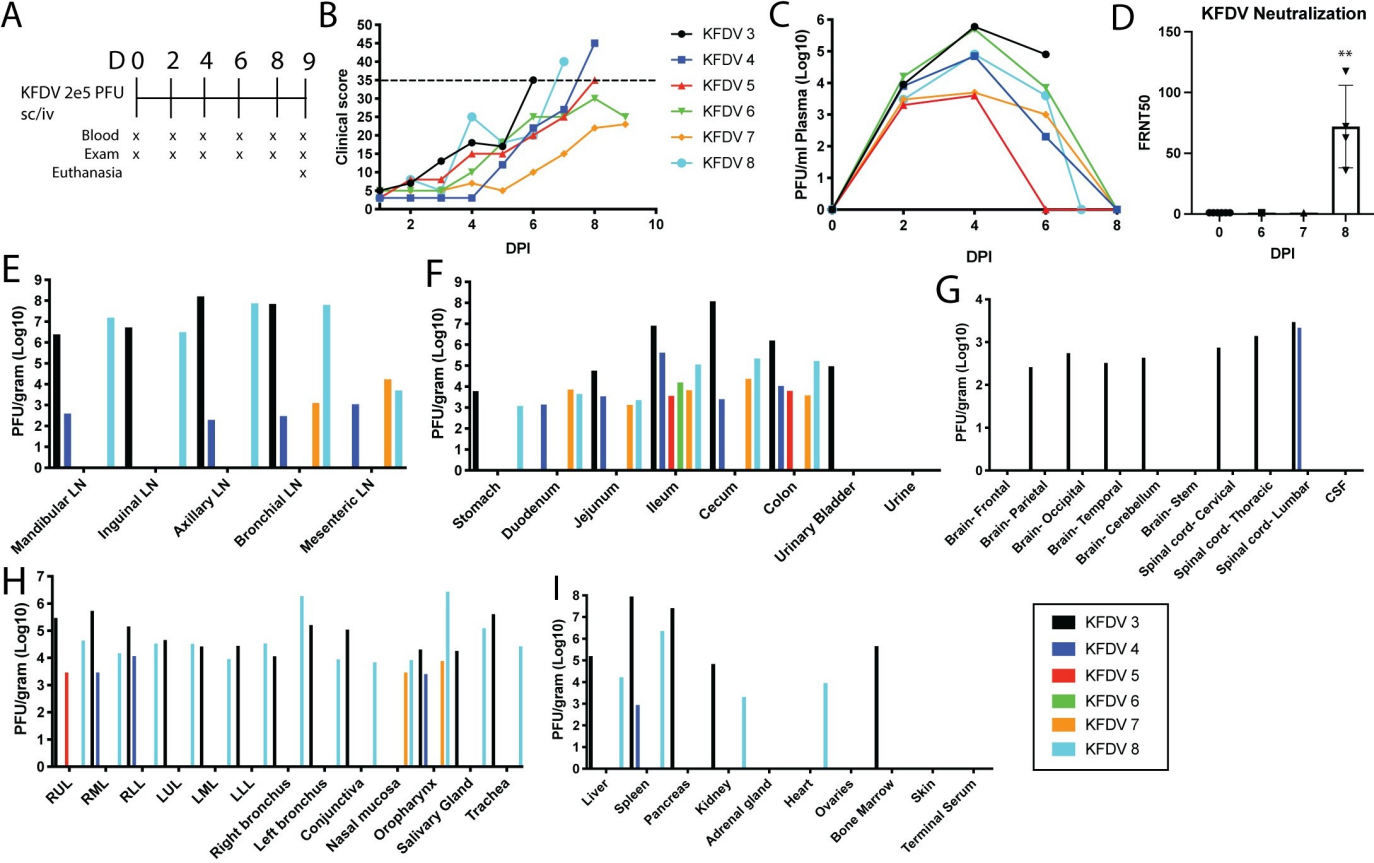
KFDV causes moderate to severe clinical illness in pigtailed macaques, and virus is present in multiple tissues. Six PTMs (KFDV 3–8) were infected with 2 x 10^5^ pfu KFDV P9605 sc/iv (10^5^ pfu, per route). **A.** Exam schedule following infection with KFDV. **B.** Animals were scored daily for clinical signs following infection. The dotted line indicates a score of 35. **C.** KFDV viremia was measured by limiting dilution plaque assay from plasma on the indicated days post infection. **D.** Neutralization assays were performed using heat-inactivated serum collected at 0, 6, 7, and 8 dpi by a focus reduction neutralization assay. FRNT50 values are reported. Error bars represent the standard deviation (SD) across four animals. Statistics were performed using an ordinary one-way ANOVA, comparing the FRNT50 values at D0 to D8 (*N* = 4; **P < 0.005). **E-I.** At terminal endpoints, or 9 dpi, animals were euthanized and virus was isolated from various tissues by plaque assay. Plaque counts were normalized to 1 gram of tissue. Data from each individual animal are plotted.

Due to the variable clinical signs and viremia between individual animals in the pilot study, an additional study was conducted using a combination of subcutaneous and intravenous (sc/iv) inoculation with KFDV P9605 (10^5^ pfu per route for a total of 2 x10^5^ pfu) in six PTMs (KFDV 3–8) (Fig 1). Clinical signs included severely decreased appetite, piloerection, flushed appearance, dehydration, and cyanotic mucous membranes (Table 2). Epistaxis was observed in 5 out of 6 animals, starting at 6 dpi and continuing through the end of the experiment. Two animals (KFDV 3 and KFDV 4) had irregular breathing patterns observable from 4 dpi. Four of the six animals met the pre-determined euthanasia criteria of a clinical score of 35 or greater between 6–8 dpi. The clinical scoring of the remaining two animals did not reach a score of 35 and was beginning to resolve at 9 dpi, prompting euthanasia for virus isolation from tissues.

**Table 2:** KFDV and AHFV clinical observations. Pigtailed macaques were observed for clinical signs following sc/iv inoculation of KFDV or AHFV. Some parameters (mucous membrane color, dehydration, breathing quality, CRT, and temperature) were only measured during exams. The presence or absence of a clinical sign are reported as Y (yes) or N (no).

	KFDV 3 (sc/iv)	KFDV 4 (sc/iv)	KFDV 5 (sc/iv)	KFDV 6 (sc/iv)	KFDV 7 (sc/iv)	KFDV 8 (sc/iv)	AHFV 1 (sc/iv)	AHFV 2 (sc/iv)	AHFV 3 (sc/iv)	AHFV 4 (sc/iv)	Frequency (sc/iv) n = 10
Decreased appetite	Severe	Severe	Severe	Severe	Decreased	Severe	Decreased	Decreased	Decreased	Severe	100%
Loss of interest in treats	Y	N	Y	Y	N	Y	N	N	N	Y	50%
Piloerection	Y	N	Y	Y	Y	Y	Y	N	N	Y	70%
Hunched posture	N	Y	Y	Y	Y	N	Y	N	N	N	50%
Loose stool	N	N	N	N	N	N	N	N	N	N	0%
No feces	Y	N	N	N	N	N	N	N	N	N	10%
Clear nasal discharge	N	N	N	Y	N	Y	N	N	N	N	20%
Transient epistaxis	N	D8 heavy epistaxis	Y	Y	Y	Y	Dried blood on nose	Y	Y	N	80%
Unsteady	N	Y	N	N	N	Y	N	N	N	N	20%
Slow and careful movements	N	Y	Y	N	N	Y	Y	N	N	N	40%
Reluctant to move	Y	N	Y	N	N	N	N	N	N	N	20%
Using cage for support	Y	Y	N	N	N	Y	Y	N	N	N	40%
Mucous membranes	bluish D6	bluish D6-8	bluish D6, D8	N	a little blood on gums	D4-7 Muddy	N	N	N	D8 Pale pink muddy	100%
Dehydration	D6 5–8%	N	5–10%	D4-6 5–8%, D9 8–10%	D9 5–8%	D4-7 5–8%	D6 5–8%, D9 10–12%	D4-9 5–8%	D6-9 10–12%	D4 5–8%, D8 10%	90%
CRT 2 seconds or more	D6, 3 sec.	D8, 2 sec	N	N	N	N	N	N	N	N	20%
Temperature change > 1°C	N	Temp drop 1.6°C D8	Temp drop 1.7°C D8	N	Temp drop 1.5°C D9	N	Temp drop 1.6°C D9	Temp drop 1.2°C D9	Temp drop 1.7°C D8	Temp drop 1.7°C D8	70%
Flushed Appearance	D6	N	D6-8	D6	N	D6-7	D6-8	N	D4-8	D7-8 pale/green	70%
Mild Facial edema	N	N	N	N	N	N	N	N	Y	N	10%
Other Comments				Liver palpable D6-9							
Score 35	D6	D8	D8	N	N	D7	D9	N	N	N	50%

All six animals had demonstrable viremia with infectious virus isolated between 2–6 dpi, with peak titer at day 4 (Fig 1C). Neutralizing antibodies were detectable in serum at 8 dpi (Fig 1D). Neutralizing activity was not detected in terminal sera from KFDV 3 and KFDV 8, that reached euthanasia criteria at 6 and 7 dpi, respectively. All oral swabs taken on exam days were negative for KFDV. However, infectious virus was isolated from rectal swabs in two out of six animals on days 4 and/or 6 post infection suggesting virus shedding from the gastrointestinal tract.

Infectious virus was readily detectable by plaque assay from multiple tissues at the time of necropsy (Fig 1E–1I). Consistently, virus was recovered from the gastrointestinal tract from all animals inoculated with KFDV. Infectious virus was recovered from the lungs, lymph nodes, and oropharynx in four of six animals. Two animals (KFDV 3 and KFDV 8) had virus present in almost every tissue examined. However, infectious virus was typically not present in the CNS, except for two animals that had virus present in the brain and spinal cord (KFDV 3) or the lumbar spinal cord (KFDV 4). Thus, PTMs infected with KFDV develop clinical illness with highest infectious virus burden in the gastrointestinal tract and lymphoid tissues from 6–9 dpi, consistent with viral shedding detected in rectal swabs. Importantly, virus tropism to the gastrointestinal tissues in the PTM model accurately reflects human clinical disease, where gastrointestinal symptoms are a hallmark sign of KFDV infection [2, 3, 31, 32].

### Clinical signs and tissue distribution of virus in AHFV-infected PTMs

**Fig 2:**
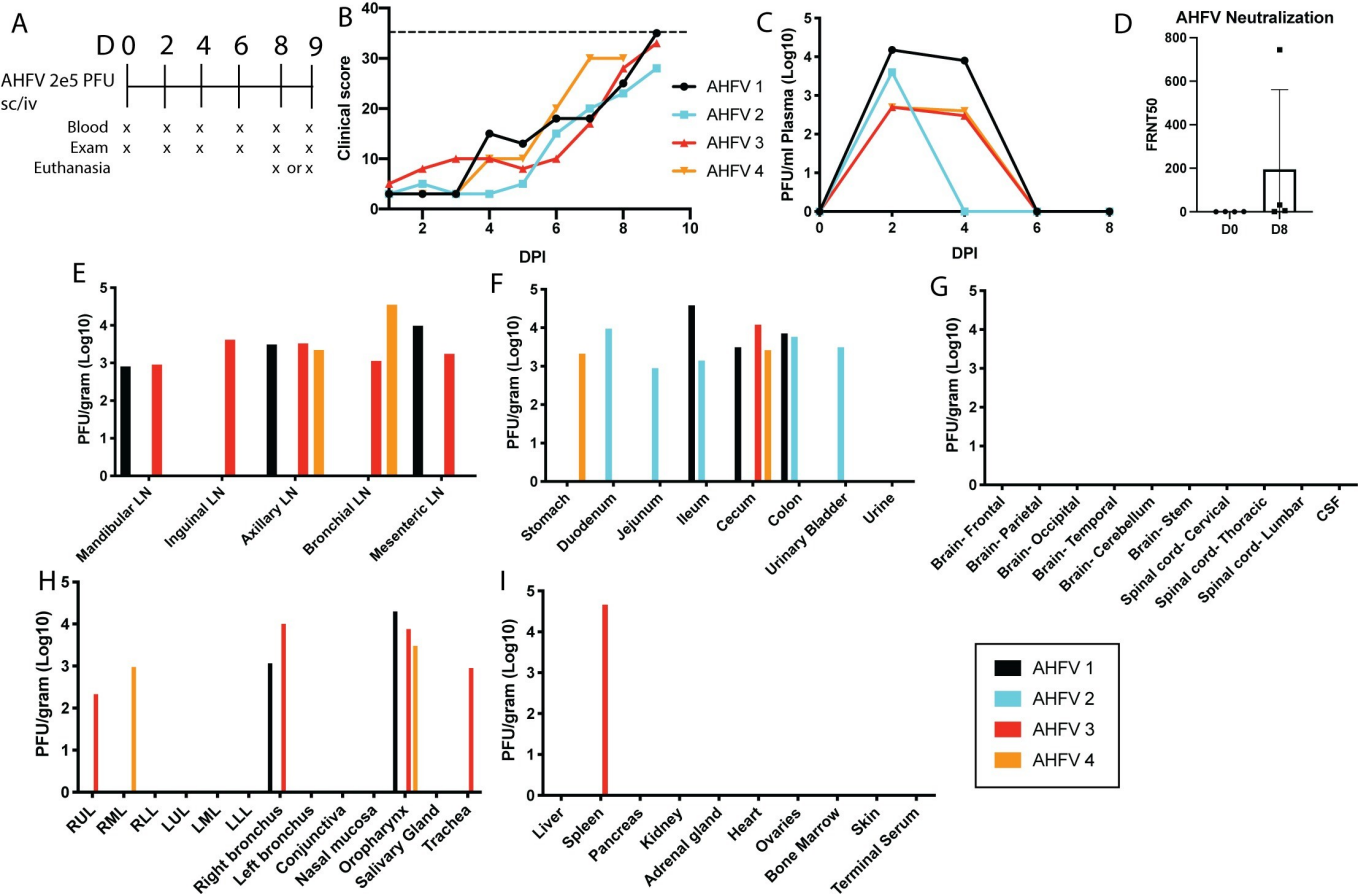
Pigtailed macaques are susceptible to disease caused by AHFV. Four pigtailed macaques (AHFV 1–4) were infected with 2 x 10^5^ pfu AHFV 200300001 sc/iv and monitored for clinical illness. **A.** Schedule for blood draws and exams in AHFV-infected animals. **B.** Clinical scores in four pigtailed macaques following infection with AHFV. Dotted line represents a score of 35. **C.** Viremia was measured in plasma over the first 8 days of infection by plaque assay. **D.** Neutralization assays were performed using a focus reduction neutralization assay. FRNT50 values are reported. Error bars show the SD across four animals. Statistics were performed using an ordinary one-way ANOVA (*N* = 4, *P < 0.05). **E-I.** At day 8 or 9 post infection, virus present in the tissue homogenate was measured by limiting dilution plaque assay, and values were normalized to 1 gram of tissue. Data from each individual animal are plotted.

AHFV is a genetic variant of KFDV, and causes similar hemorrhagic disease in humans although its pathogenesis has not been examined in NHP models. Therefore, we extended the model by inoculating four PTMs with AHFV 200300001 by the sc/iv route, followed by monitoring for clinical signs with exams and blood draws every two days (Fig 2A). Animals inoculated with AHFV developed disease signs that resembled infection with KFDV, including flushed appearance, dehydration, and transient epistaxis (Table 2). One out of four animals reached a clinical score of 35 on day 9 post infection, while other animals had a maximum clinical score of 28 to 33 (Fig 2B). As it was clear from clinical parameters that the disease was resolving in the remaining three animals, all PTMs were euthanized at 8 or 9 dpi to examine virus tissue distribution (Fig 2E–1I).

All four animals had detectable viremia between 2 and 4 dpi (Fig 2C) and developed low levels of neutralizing antibodies by 8 dpi (Fig 2D). However, all rectal swabs taken on exam days were negative for infectious virus. The oral swab of AHFV 4 was positive on day 4 post infection, but all other oral swabs were negative. Despite a lack of shedding, AHFV could be isolated from the gastrointestinal tract in all four animals, similar to KFDV (Fig 2F). Three out of four animals also had virus in the lymph nodes, oropharynx, and lungs. No virus was detectable in the brain, CSF, or spinal cord (Fig 2E–2I). Thus, PTMs develop clinical signs of illness and virus can be isolated from multiple tissues following inoculation with either KFDV or AHFV.

### Changes in serum inflammatory markers and clinical blood parameters following infection with KFDV and AHFV

**Fig 3:**
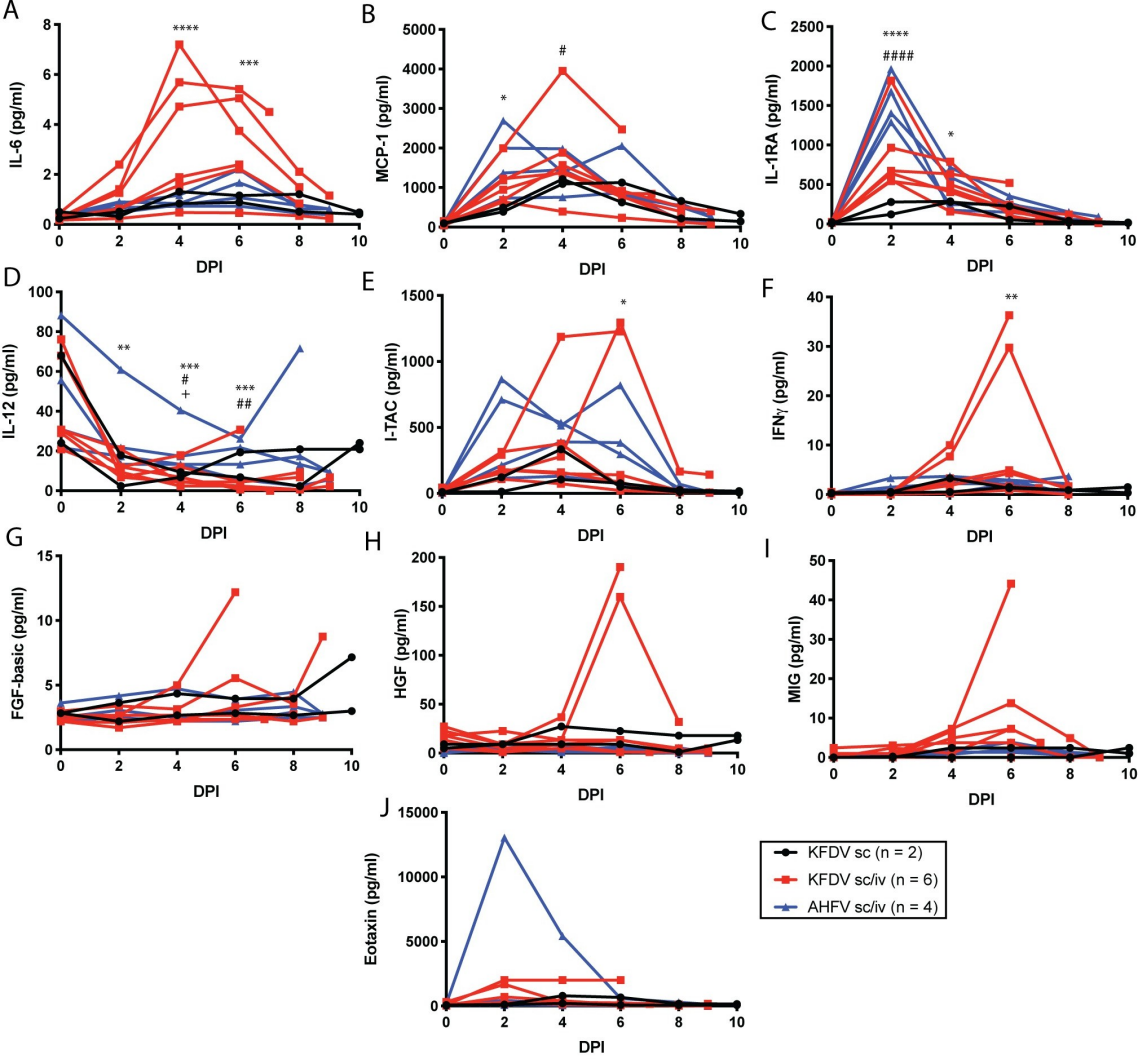
Cytokine and chemokine analysis in serum of KFDV and AHFV-infected animals. Pigtailed macaques were infected with KFDV by the sc or sc/iv route, or AHFV by the sc/iv route. The number of animals in each group is indicated in the legend. Serum was isolated from blood samples obtained during clinical exams at the indicated days post infection. Cytokine and chemokines were measured from sera using a 29-plex Monkey cytokine detection kit. The following parameters are included: **A.** IL-6, **B.** MCP-1, **C.** IL-1RA, **D.** IL-12, **E.** I-TAC, **F.** IFNγ, **G.** FGF-Basic, **H.** HGF, **I.** MIG, **J**. Eotaxin. Statistics were performed by comparing the change in the parameter compared to the D0 measurement using Sidaks multiple comparison test. Significance symbols are represented as follows: KFDV sc/iv *, AHFV sc/iv #, KFDV sc +. (KFDV sc/iv *N* = 6, AHFV sc/iv *N* = 4, KFDV sc *N* = 2; *P < 0.05, **P < 0.005, ***P < 0.0005, ****P < 0.0001.

To measure the changes in inflammatory biomarkers following inoculation with KFDV or AHFV, serum cytokine and chemokines were measured throughout infection. Significant increases were observed with IL-6, MCP-1, IL-1RA, I-TAC, and IFNγ in the KFDV sc/iv group (Fig 3A–3F), with peaks at 2, 4, or 6 dpi. The same parameters were elevated in AHFV-infected animals, but only MCP-1 and IL-1RA reached statistical significance. Both KFDV sc/iv and AHFV inoculated animals had a significant decrease in IL-12 (Fig 3D). Other cytokines such as FGF-Basic, HGF, MIG, and Eotaxin were not uniformly altered within groups, but specific animals experienced changes in those cytokines following infection (Fig 3G–3J). Mild changes in the reported cytokines and chemokines were also observed in the KFDV sc group, including reduced IL-12 compared to baseline. Statistics were not performed after day 6 due to the decreasing group sizes in the KFDV and AHFV groups. Cytokine dynamics have not been described in sera from human patients with confirmed KFDV or AHFV infection or in NHPs inoculated with KFDV. A similar set of cytokines were reported elevated in the KFDV mouse model. In mice, IL-6, IL-10, IFNγ, and MCP-1 were increased while TNF-α was reduced in the spleen, and these cytokine dynamics were more limited in mice infected with AHFV [11].

**Fig 4:**
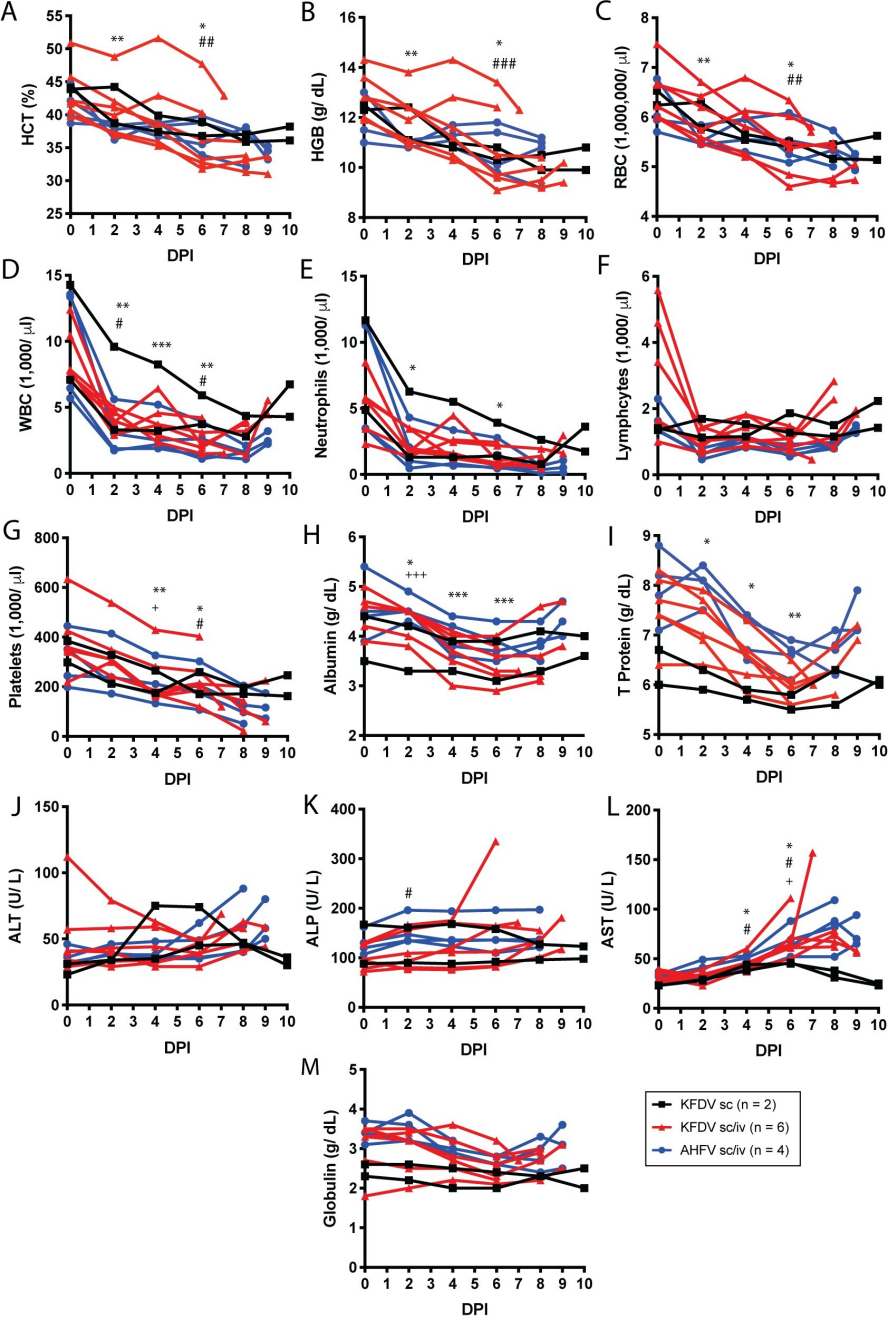
Blood parameters in pigtailed macaques infected with KFDV or AHFV. Blood was collected during exams as indicated. Complete blood counts were analyzed from EDTA blood (A-G), and blood chemistries were measured from sera (H-M). **A**. Hematocrit (HCT), **B**. Hemoglobin (HGB), **C.** Red blood cells (RBC), **D.** White blood cells (WBC), **E.** Neutrophils, **F.** Lymphocytes, **G**. Platelet counts, **H.** Albumin, **I**. Total protein, **J**. Alanine aminotransferase (ALT), **K**. Alanine phosphatase (ALP), **L**. Aspartate transaminase (AST), **M**. Globulin. Statistics were performed by comparing the change in the parameter at day 2, 4, and 6 compared to the day 0 measurement using Dunnett’s multiple comparison test. Significance symbols are represented as follows: KFDV sc/iv *, AHFV sc/iv #, KFDV sc +. (KFDV sc/iv *N* = 6, AHFV sc/iv *N* = 4, KFDV sc *N* = 2; *P < 0.05, **P < 0.005, ***P < 0.0005, ****P < 0.0001).

Within the hemogram, all groups showed a decrease in HCT, HGB, and RBC throughout the study (Fig 4A–4C). In some animals, these decreases resulted in a non-regenerative anemia particularly within the KFDV sc/iv group. Similarly, within the leukogram, all groups had a decrease in leukocytes that was driven by significant decreases in both neutrophils and lymphocytes (Fig 4D–4F). The majority of animals had leukopenia, neutropenia and lymphopenia, between D2–8 with some signs of recovery prior to the endpoint. These findings are consistent with a previous study within bonnet macaques (*Macaca radiata*) [19]. Animals in all groups demonstrated decreases in platelets throughout the study as well with some animals in the KFDV sc/iv and AHFV sc/iv becoming thrombocytopenic (<100 K/ul) (Fig 4G). The combination of the leukopenia and thrombocytopenia is a hallmark of human infection with KFDV [26], but has not been observed in other NHP models of KFDV pathogenesis.

Although reported in humans and bonnet macaques that liver enzymes increase with disease, this was not as clear within this model. Only a slight trend of increasing ALT and AST towards later timepoints was noted following AHFV sc/iv or KFDV sc/iv inoculation (Fig 4J–4L), although these increases are not considered clinically relevant. More consistently, decreased albumin, total protein and globulin were observed up to D6 with signs of recovery after that timepoint (Fig 4H, 4I and 4M). Three KFDV sc/iv animals and one KFDV sc animal had hypoalbuminemia (< 3.5 g/dl). The mouse model of KFDV has also been reported to show hypoalbuminemia in the later stage of disease [12]. Taken together, the prolonged viremia, increased tissue burden, and upregulation of specific cytokines suggests that inoculation with KFDV results in more severe disease compared to AHFV in PTMs.

### Histopathological findings in KFDV- and AHFV- infected PTMs

Gross pathology from KFDV- and AHFV- inoculated animals at necropsy is listed in Table 1. All six KFDV sc/iv animals had pale or discolored livers. Two animals had hyperemic lungs with one animal having congested lungs and foam in the trachea. In the AHFV sc/iv group, two animals had enlarged spleens and/or livers, and two had dried blood in the nasal cavity. AHFV 1, the individual reaching a clinical score of 35, had hemorrhages present on the liver and lungs. Thus, PTMs can be used to model both KFDV and AHFV disease, with clinical signs closely resembling human infection.

Despite some animals reaching the pre-established euthanasia criteria, it was surprising that no major histopathological findings could be definitively attributed to KFDV or AHFV. In the KFDV sc/iv group, KFDV 4–8 had a chronic, marked-severe fatty infiltration of the liver parenchyma. KFDV 3–6 had minimal-mild amounts of lymphocytic infiltrates within the lamina propria of intestinal tract. In the AHFV sc/iv group, AHFV 1 and 4 had a chronic, marked-severe fatty infiltration of the liver parenchyma, and AHFV 3 had mild amyloidosis of the liver. AHFV 1 had a marked neutrophilic bronchopneumonia located in the left middle lung lobe and a minimal lesion in the left upper lobe. AHFV 4 had multifocal mucosal necrosis of the ileum. Within the KFDV sc group, KFDV 2 had moderate amyloidosis of the liver and minimal to mild lymphocytic infiltrates within the lamina propria of the intestinal tract. However, it is possible that these conditions are associated with chronic illness due to age and history of these individuals, and not directly due to virus infection.

### In-situ hybridization and Immunohistochemistry

**Fig 5:**
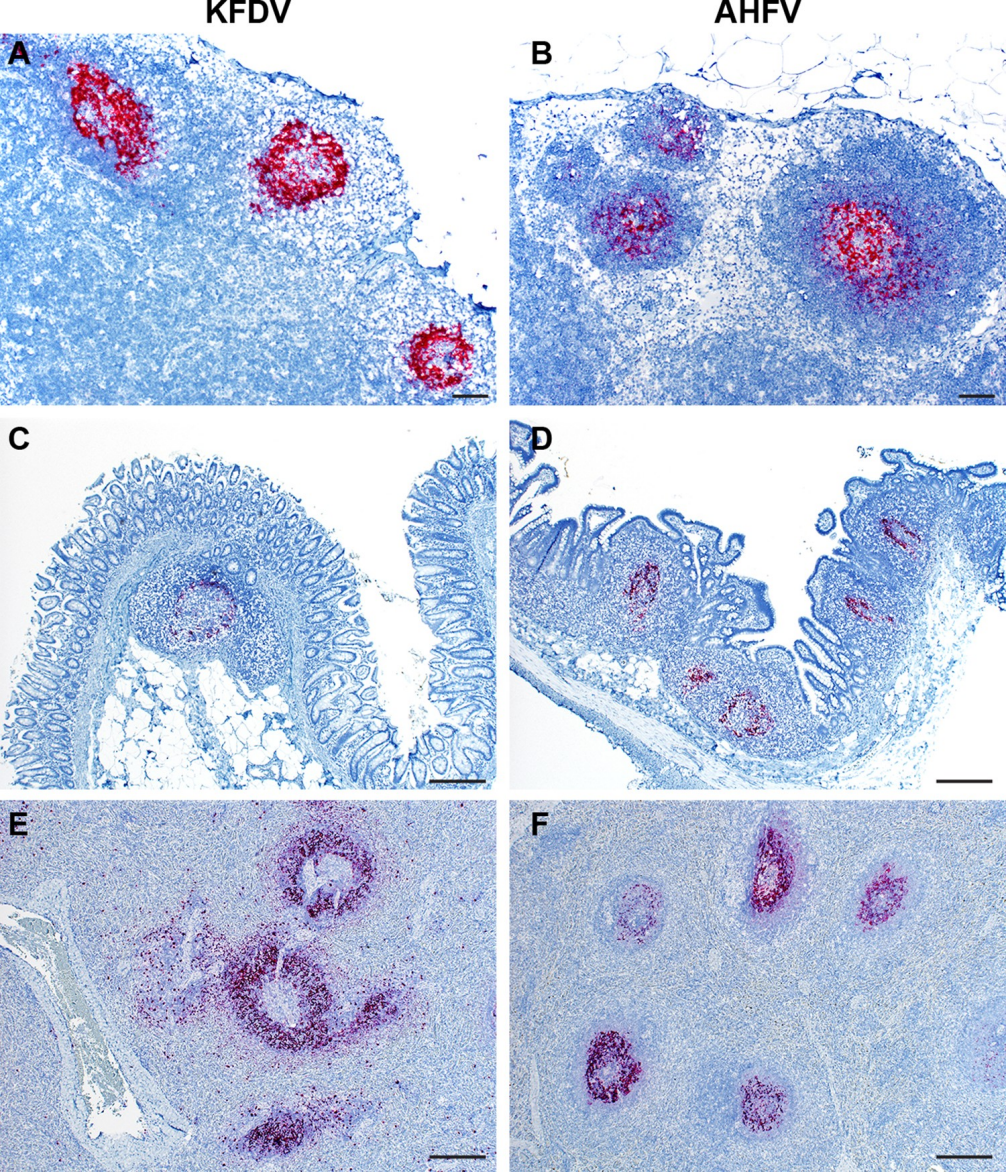
KFDV and AHFV RNA detection by ISH in the lymph node, intestine, and spleen of infected animals. A KFDV RNAscope probe was used to detect viral RNA from tissue sections derived from either KFDV or AHFV-infected PTMs. Tissues from KFDV sc/iv infected animals (**A, C, E**) or AHFV sc/iv infected animals (**B, D, F**) were examined. Representative images are displayed for **A, B.** mesenteric lymph node (40X, bar = 200 μm); **C.** cecum (100X, bar = 50 μm), **D**. ileum (100X, bar = 50 μm), and **E, F.** spleen (100X, bar = 50 μm).

In situ hybridization (ISH) was performed using a 20-nucleotide probe targeting KFDV NS4B and NS5. The target region of this probe in the KFDV P9605 genome is 93% identical to AHFV (200300001), and detected AHFV genome. Within the KFDV sc inoculated group, only the bronchial lymph node of KFDV 1 showed positivity. However, in the KFDV sc/iv and AHFV sc/iv groups, immunoreactivity was observed in gut-associated lymphoid tissue (GALT), splenic, mediastinal, and mesenteric lymph node follicles (Fig 5). Consistent with the virus isolation data, KFDV positivity was also observed in the bronchus- associated lymphoid tissue (BALT) (S3A Fig). These data indicate that viral RNA in both KFDV and AHFV-inoculated animals is frequently associated with lymphoid tissue at the most acute stages of disease. IHC was performed in the brain of KFDV 3 since infectious virus was directly isolated from brain samples of only this animal (Fig 1G). KFDV 3 had a focus of positivity in a cluster of mononuclear cells in the choroid plexus (S3B Fig).

**Fig 6:**
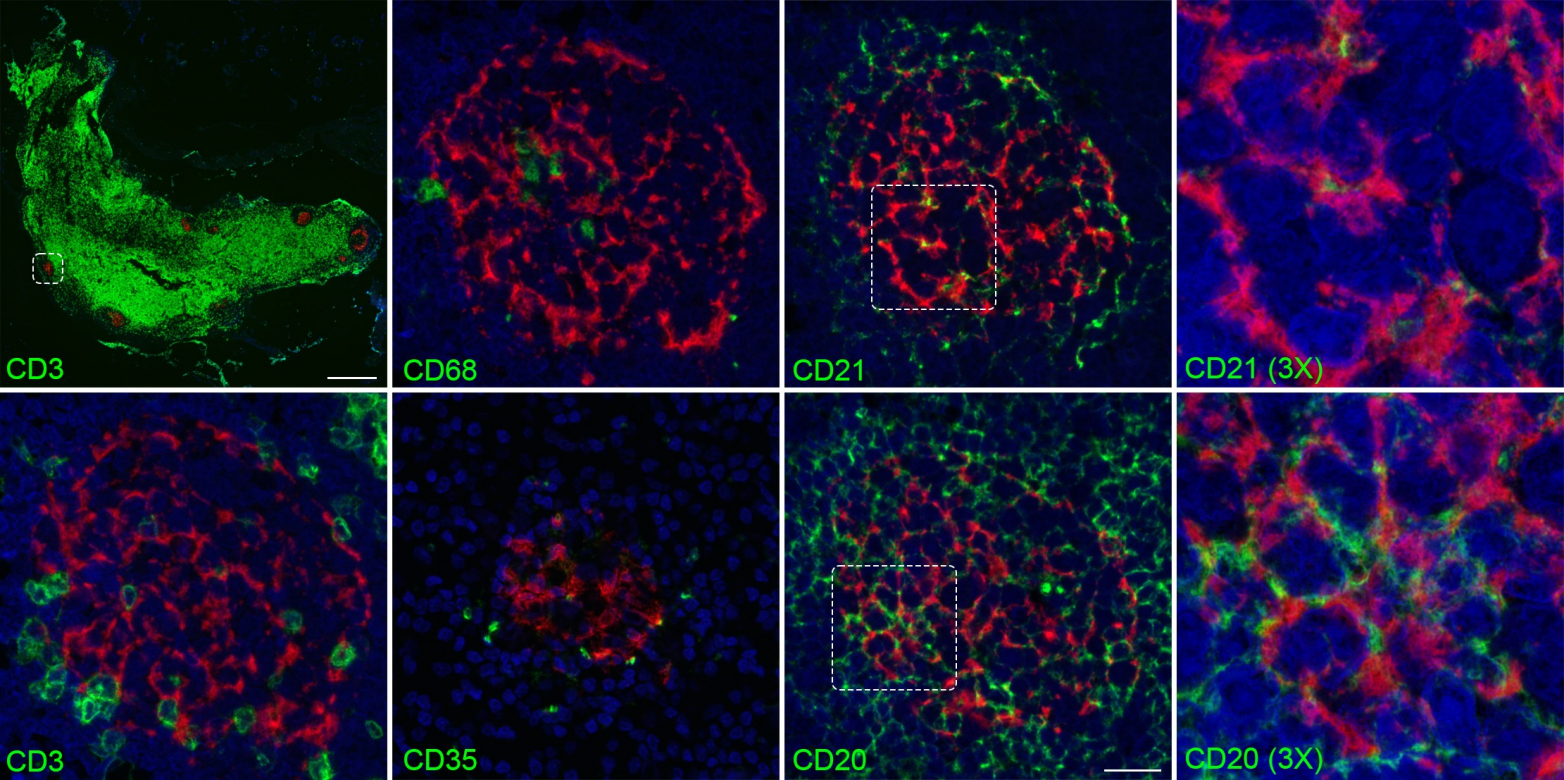
Mesenteric lymph node double staining with KFDV and CD3, CD20, CD21, CD35, or CD68. Mesenteric lymph node sections from an animal infected sc/iv with KFDV was subjected to RNAscope and IHC for CD3, CD20, CD68, CD21, and CD35 using the RNAscope VS Universal ISH-IHC HRP fluorescent assay. KFDV (red) and cell markers (green). Scale bar for low-magnification CD3/KFDV panel, 500 μm; for high-magnification images of CD3, CD20, CD68, CD21, and CD35, 20 μm.

Lymphoid tissues were examined by ISH combined with IHC to directly identify cell types commonly associated with viral RNA positivity. Mesenteric lymph nodes were probed with antibodies directed towards CD3+, CD20+, CD68+, CD21+, and CD35+ cells for detection of T cells, B cells, macrophages, and follicular dendritic cells respectively. KFDV RNA did not colocalize with CD3+ T cells. However, CD20+ B cells were closely associated with KFDV RNA (Fig 6) and AHFV RNA (S4 Fig). CD21+ and CD35+ follicular dendritic cells were in near proximity to the KFDV+ cells, but did not colocalize. CD68+ macrophages, a known flavivirus target [33, 34], were also associated with KFDV RNA in some lymph nodes, such as the mediastinal lymph node (S5 Fig). KFDV RNA association with CD20+ cells may either be direct infection of B cells, or the virus may be trapped on surface of B cells and/or follicular dendritic cells. Thus, the IHC and ISH provides evidence of infection in macrophages and strong association with B cell follicles, with no obvious differences between KFDV and AHFV.

### Transcriptomic characterization

**Fig 7:**
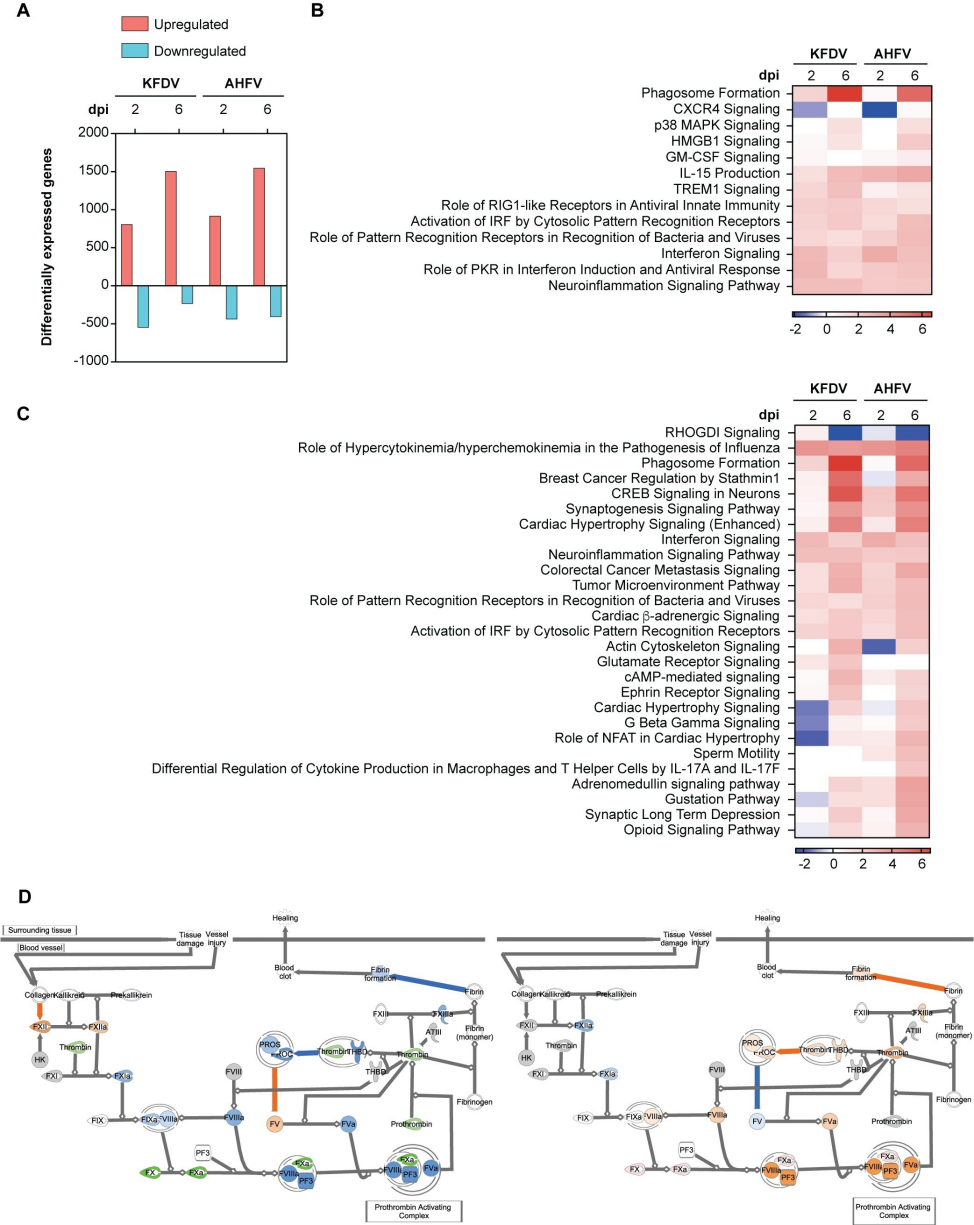
Transcriptional dynamics in blood of PTMs inoculated with KFDV and AHFV. Whole blood from PTMs inoculated sc/iv with KFDV or AHFV was subject to RNAseq at 0, 2, and 6 dpi. **A.** Differentially expressed genes (DEG) at 2 and 6 dpi with fold change > |1.5| relative 0 dpi and *P* < 0.05. **B.** Heat map of z-scores depicting changes in cellular immune response pathways following inoculation with KFDV or AHFV. **C.** Heat map of z-scores showing significantly enriched and differentially activated pathways between KFDV and AHFV- inoculated PTMs. **D.** Predicted function of the intrinsic prothrombin activation pathway at 2 dpi using IPA following inoculation with KFDV (left) and AHFV (right). (Predicted activation (orange), predicted inhibition (blue), observed downregulation (green).

We assessed systemic host responses to infection using RNA sequencing to characterize the overall global transcriptome in whole blood collected at 0, 2, and 6 dpi. Differentially expressed genes (DEG) were defined as those with fold change > |1.5| relative to the baseline at 0 dpi and *P* < 0.05 (Fig 7A). To better understand the character of the immune response, we first limited our functional analysis to signaling pathways associated with immune function that were significantly enriched (adjusted *P* > 0.05) with a z-score > |2|. IPA z-scores predict pathway activation or inhibition and are calculated using DEGs in a given pathway. We observed that both KFDV and AHFV induced robust antiviral responses, including both interferon responses and inflammatory responses, beginning at 2 dpi and generally sustained through 6 dpi (Fig 7B). This suggests that the two viruses induce similar host innate antiviral responses. We also compared all significantly enriched and differentially activated pathways to identify unique features of the host response to infection with either virus (Fig 7C). This demonstrated clear early inhibition in pathways associated with cardiovascular function in the PTMs infected with KFDV but not in animals infected with AHFV, as well as a failure to induce pathways associated with macrophage function and T cell differentiation. To investigate the possible mechanism underlying KFDV coagulopathy, we used the IPA Molecular Activity Predictor tool to compare predicted function of the intrinsic prothrombin activation pathway at 2 dpi (Fig 7D). In KFDV-infected PTM, we observed both transcriptional downregulation of key clotting factors, as well as a predicted inhibition of other procoagulant molecules, indicating a possible impairment of normal coagulation. In AHFV-infected PTMs, we observed upregulated factor X, as well as predicted activation of numerous clotting factors including thrombin, prothrombin, and fibrin formation. Thus AHFV-infected PTMs retain function of normal coagulation compared to KFDV-infected PTMs. While the innate transcription profiles to infection were similar, the coagulation gene expression profiles may provide insight into differences in pathogenesis between KFDV and AHFV in PTMs.

## Conclusion

The emergence of AHFV in the Arabian Peninsula and the increasing geographical range of KFDV in India highlights the urgent need for relevant animal models for testing vaccines and therapeutics. In this study, PTMs were tested for susceptibility and disease following infection with KFDV or AHFV. We found that animals inoculated by the sc/iv route demonstrated moderate to severe disease with KFDV and AHFV. KFDV infection in humans is commonly characterized by a sudden onset of fever, muscle weakness, back ache, vomiting, diarrhea, headache, reddened eyes, and some hemorrhagic manifestations such as bleeding from gums, or recurring epistaxis [31]. In rare cases, the bleeding can be more severe, with hematemesis and hematochezia, and/or hemorrhage in the lungs. Consistent with case reports in humans, PTMs inoculated with KFDV had clinical signs such as weakness, loss of appetite, flushed appearance, epistaxis, and dehydration. Clinical signs were similar but less severe after infection with AHFV. One major advantage of the PTM model over current models is that these animals experienced marked thrombocytopenia and hemorrhagic manifestations following inoculation with KFDV and to a lesser extent AHFV. In the KFDV group, most animals experienced epistaxis during the acute phase, except one animal that reached the euthanasia criteria at an early time point. Two animals inoculated with KFDV had hyperemic lungs, and one animal inoculated with AHFV had hemorrhages in the lung and liver. Together, the PTM model for KFDV and AHFV closely resembles human infection and can be used for exploration into mechanisms of KFDV and AHFV pathogenesis. Mechanisms underlying KFDV-induced hemorrhage have been postulated to result from either direct infection of endothelial cells or indirect activation of endothelial cells via cytokine release from infected dendritic cells and macrophages [35]. However, RNAseq of whole blood from our study suggests that animals infected with KFDV had an early suppression of clotting factor gene expression which combined with marked thrombocytopenia likely explains hemorrhage. In contrast, DEGs in AHFV-infected PTMs at 2 dpi included upregulation of clotting factors which may explain why KFDV induces more severe disease than AHFV in the PTM model.

Infectious virus was frequently isolated from the gastrointestinal tissues, lungs, and lymph nodes in PTMs inoculated with KFDV or AHFV. In-situ hybridization revealed that the infection at these sites was associated with lymphoid tissue, particularly in the GALT, BALT, and the B cell zone within lymph nodes. These results are similar to reports of KFDV-infected bonnet macaques where antigen was seen in lymphocytes in the spleen as well as lymphoid cells in the gastrointestinal tract in KFDV-infected animals [18]. However, bonnet macaques inoculated with KFDV showed virus positivity in epithelial cells within the gastrointestinal tract, but this was not observed in tissues from infected PTMs. Virus infection within lymphoid tissue such as the GALT or the BALT in NHPs has been shown with other viruses known to infect lymphocytes such as measles virus, and HIV [36, 37]. Within lymphoid tissue, virus positivity in B cell follicles has been observed in other viral infection models including Zika virus-infected rhesus macaques, and in SIV-infected rhesus macaques [38, 39]. This phenomenon could be evidence of trapped virus within B cell zones, possibly associated with follicular dendritic cells. While monocytes/macrophages are known targets of flavivirus infections, the consequences of potential B cell infection or antigen positivity is not described. A recent report of human infection with KFDV showed decreased levels of activated B cells in the acute phase of illness [40]. The association of virus and B cells in the PTMs could provide mechanism for this phenomenon. B cell activation was not measured in the current study, but the KFDV-infected PTMs developed neutralizing antibodies associated with the resolution of viremia at 7–9 dpi, suggesting that the B cells were functional in this model.

KFDV is considered primarily a viscerotropic disease, where the second disease phase with CNS involvement is rare in humans [3, 31]. Approximately three weeks following the acute phase, a second phase of illness may occur in roughly 10–20% of cases, characterized by recurrence of fever, headache, tremors, and neck stiffness [31, 32, 41]. The current study examined KFDV and AHFV in PTMs up to 9 dpi in the animals inoculated sc/iv when the primary disease was clearly resolving. Future work could extend the observation period to 4–5 weeks post infection to determine the incidence of this secondary phase of disease in PTMs. However, KFDV was found in the brain of only one animal euthanized at 6 dpi, suggesting that virus localization in the brain may be an uncommon event during the acute phase of disease. In contrast with the PTM model, KFDV is commonly isolated from the brain of infected bonnet macaques, even at acute time points [18–20]. Human case reports with AHFV show central nervous system manifestations, including encephalitis, occurring in about 20–25% of patients [7, 42]. AHFV was not detected in the CNS in inoculated PTMs, which may reflect differences between the PTM model and human cases. However, this study characterizes the acute phase of infection in only four AHFV-infected PTMs. Future work may expand the duration of infection to determine if AHFV migration to the CNS could occur at a later time post infection. It should be noted that the animals used in this study were all females, 10–16 years old. Generally these animals were in good health for their age, with no traditional chronic issues such as diabetes or other endocrinopathies. Additional studies will be necessary to determine if sex or age could influence the infection outcome.

In this work, we established that PTM TRIM5 does not block infection of KFDV, further providing rationale to characterize the PTM model for KFDV pathogenesis. Compared to previous reports in rhesus macaques, KFDV appears to cause more severe disease in PTMs. While this is consistent with the pattern of TRIM5 restriction, it is unknown whether additional barriers to KFDV and AHFV replication exist in the PTM model. In development of the HIV-1 PTM pathogenesis model, it was necessary to overcome innate immune barriers such as APOBEC3, tetherin, and MX2 [26, 43, 44]. Future investigation will determine what additional innate immune barriers to infection may exist for KFDV and AHFV, as was found with HIV-1.

One aspect of KFDV and AHFV biology that was not addressed in this model was the ability of tick saliva to enhance dissemination and disease. Tick saliva contains a mixture of many factors known to modulate innate and adaptive immune responses (reviewed in [45, 46]). Tick saliva enhances TBFV transmission, dissemination, and impacts disease in sensitive animals [47–56]. Tick saliva has not been used in TBFV NHP models, but its use may improve model development as it is clear from our pilot experiment with the sc route that the skin represents a strong barrier to systemic infection that was overcome by iv inoculation in order to develop PTMs as a disease model.

While no antiviral drugs are available to treat TBFV infections, several vaccines have been developed against TBFVs. The current vaccine for KFDV is a formalin-inactivated virus preparation that requires multiple boosters and has limited efficacy. Unfortunately, patients who received the KFDV vaccine may still develop viremia and clinical illness after KFDV infection [40, 57, 58]. While the TBEV vaccine elicits cross-neutralizing antibodies that may protect against KFDV and AHFV [59], the TBEV vaccine is not approved for prevention of disease caused by these hemorrhagic TBFVs. The PTM model of KFDV and AHFV pathogenesis will be useful for future efforts in vaccine and antiviral development, as well as further examining mechanisms of virus pathogenesis.

## Methods

### Ethics statement

All work with infectious TBEV, KFDV, and AHFV was conducted at Biosafety Level 4 facilities at the Integrated Research Facility at the Rocky Mountain Laboratories (Hamilton, MT). All experiments involving PTMs were performed in strict accordance with the approved Rocky Mountain Laboratories Animal Care and Use Committee protocol 2018-066-E and following recommendations from the Guide for the Care and Use of Laboratory Animals of the Office of Animal Welfare, National Institutes of Health and the Animal Welfare Act of the US Department of Agriculture, in an Association for Assessment and Accreditation of Laboratory Animal Care International (AAALAC)-accredited facility.

### Pigtailed macaques

Twelve female PTMs aged 10 to 16 years of age were housed in a humidity, temperature, and light controlled facility in adjacent cages for social interaction. Animals were closely monitored at least twice daily by trained personnel. Animals were fed a commercial monkey chow twice per day, and their diets were supplemented with a variety of fruits, vegetables, and treats. Water was available ad libitum. Animals were provided with manipulanda, human interaction, and audio and visual enrichment. Virus inoculations were performed with the indicated virus diluted in sterile PBS. For subcutaneous (sc) infections, 1x10^5^ pfu was delivered in a single injection between the shoulder blades (1 ml volume). Intravenous (iv) inoculations were performed with 1x10^5^ pfu diluted virus using an IV catheter (1 ml volume). Animals were closely monitored and scored twice daily after showing signs of illness. Clinical scoring was evaluated by trained veterinary staff in each category: general appearance; skin and haircoat; nose, mouth, eyes, and head; respiration; feces and urine; appetite; and activity level. To calculate the clinical score, scores from each category were totaled on the approved clinical score sheet. Animals were euthanized if they reached a score of 35 or more, which is considered critical illness based on pre-established endpoints. Exams occurred on 0, 2, 4, 6, 8, 10, 14, 21, 28, 35, and 42 or 43 dpi. At each exam, blood was drawn in EDTA and serum separation tubes, and rectal and oral swabs were collected. Hematology analysis was completed on a ProCyte DX (IDEXX Laboratories, Westbrook, ME, USA) and the following parameters were evaluated: red blood cells (RBC), hemoglobin (Hb), hematocrit (HCT), mean corpuscular volume (MCV), mean corpuscular hemoglobin (MCH), mean corpuscular hemoglobin concentration (MCHC), red cell distribution weight (RDW), platelets, mean platelet volume (MPV), white blood cells (WBC), neutrophil count (abs and %), lymphocyte count (abs and %), monocyte count (abs and %), eosinophil count (abs and %), and basophil count (abs and %). Serum chemistries were completed on a VetScan VS2 Chemistry Analyzer (Abaxis, Union City, CA) and the following parameters were evaluated: glucose, blood urea nitrogen (BUN), creatinine, calcium, albumin, total protein, alanine aminotransferase (ALT), aspartate aminotransferase (AST), alkaline phosphatase (ALP), total bilirubin, globulin, sodium, potassium, chloride, and total carbon dioxide.

### Cells and viruses

Vero E6 cells (ATCC CCL-81) and HEK293 cells (ATCC CRL-1573) were grown in Dulbecco’s modified Eagle media (DMEM) containing 10% fetal bovine serum (FBS) and 1% antibiotics Penicillin-Streptomycin in an incubator at 37°C and 5% CO_2_. KFDV (P9605) and AHFV (200300001) seed stocks were obtained from the University of Texas Medical Branch (UTMB) [11]. Virus seed stocks were propagated once on Vero cells, and virus working stocks were aliquoted and frozen in liquid nitrogen tanks. Sequencing confirmed that the AHFV virus stock had no significant snps detected relative to the published sequence JF416954.1. The KFDV virus stock has 8 snps relative to the published sequence JF416958.1: position 6690 A to G, 10551 G to A, 10563 G to A, 10579 T to C, 10580 T to C, 10599 G to A, 10676 C to T, and 10735 A to G. These SNPs resulted in one AA change in NS4A (T2187A), and the other snps were localized to the 3’UTR.

### Virus isolation

Whole blood was collected in Vacutainer EDTA Tubes and overlayed on lymphocyte separation medium (Corning). After centrifugation, the plasma layer was collected and titrated on Vero cells by limiting dilution plaque assays. For virus isolation from tissues, samples were weighed and homogenized in DMEM containing 10% FBS and 1% antibiotics using 5 mm stainless steel beads (Qiagen) and the TissueLyser II (Qiagen). For plaque assays, samples were serially diluted in DMEM and added to Vero cells for 30 minutes to 1 hr while rocking the plate every 15 minutes. The cells were overlaid with 1.5% carboxymethylcellulose (CMC) (Sigma) overlay in Minimum Essential Media (MEM). Following plaque development, plates were flooded with 10% formalin and plaques were visualized with a 1% crystal violet solution.

### Cytokine and chemokine analysis

Sera was inactivated by irradiation with 10 megarads (1 x 10^5^ Gray) according to standard biosafety protocols [60]. Cytokines and chemokines were measured from irradiated sera using a magnetic Cytokine 29-Plex Monkey Panel (ThermoFisher) according to the manufacturer’s instructions. Sera was incubated with antibody beads for 2 hours followed by addition of detection and indicator antibodies. Cytokine levels were detected using a Bio-Plex 200 System with high-throughput fluidics (Bio-Rad).

### Neutralization assays

Neutralization tests were performed on Vero cells by a focus forming reduction assay. Irradiated sera (10 megarads) were heat-inactivated for 1 hr at 56°C. Two-fold serial dilutions of sera (1/20 to 1/40760) were prepared and mixed 1:1 with media containing 50 to 100 ffu of KFDV or AHFV for a final serum dilution of 1/40 to 1/81520. Virus and antibody complexes were incubated at 37°C for two hours. The complexes were then plated onto Vero cells in black 96-well plates for 30 minutes followed by addition of CMC overlay. At 24 hpi, Vero plates were washed once with PBS and flooded with 10% formalin overnight. Plates were removed in fresh 10% formalin according to established sample removal protocols. Plates were permeabilized with 0.1% Triton-X-100 in PBS followed by an incubation in block buffer (1% Bovine serum albumin (BSA) in PBS). Foci were probed with anti-E (11H12), diluted in 0.1% BSA followed by an Alexa Flour 488-conjugated secondary antibody (Invitrogen, A32723). Foci were counted using an Immunospot S6 Universal Analyzer. FRNT50 values were calculated using nonlinear regression analysis.

### KFDV RNA detection

RNA was extracted from 140 μl plasma using the QIAamp Viral RNA Mini Kit (Qiagen). RNA was irradiated (10 megarad) for removal from the BSL4 according to established sample removal protocols [60]. Following removal, cDNA was generated using SuperScript VILO cDNA Synthesis Kit (ThermoFisher). Viral transcripts were detected by qRT-PCR using the Platinum Quantitative PCR SuperMix-UDG with ROX (ThermoFisher). Primer and probe sets used for qRT-PCR assays are as follows: KFDV forward primer: TGGCCAGCAGAGGGTTTTTA, reverse primer: AACGGCCCTCATGATGATCT, probe: CAAAGCGCAGGAGC.

### H&E

Tissues were fixed in 10% Neutral Buffered Formalin x2 changes, for a minimum of 7 days according to IBC approved protocols. Tissues were placed in cassettes and processed with a Sakura VIP-6 Tissue Tek, on a 12-hour automated schedule, using a graded series of ethanol, xylene, and PureAffin. Embedded tissues are sectioned at 5 μm and dried overnight at 42°C prior to staining.

### In Situ Hybridization (ISH) and microscopy

Chromogenic detection of KFDV viral RNA was performed on formalin fixed tissue using the RNAscope VS Universal AP assay (Advanced Cell Diagnostics Inc.) on the Ventana Discovery ULTRA stainer using an RNAscope 2.5 VS Probe—V-KFDV-PP consisting of 20 probe pairs targeting the positive sense RNA at nt 7597–8486 of KFDV (Advanced Cell Diagnostics Inc. cat# 591199). ISH was performed according to manufacturer’s instructions. Fluorescent detection of KFDV ISH and IHC was performed using the RNAscope VS Universal ISH-IHC HRP fluorescent assay (Advanced Cell Diagnostics Inc.) on the Ventana Discovery ULTRA stainer according to manufacturer’s instructions. For the IHC, primary antibodies include CD3 (clone 2GV6) rabbit monoclonal (Roche Tissue Diagnostics cat#790–4341), CD20 rabbit polyclonal (Thermo Fisher Scientific cat#RB-9013), CD68 (clone KP1) mouse monoclonal (Agilent cat#M081401-2), CD21 (Novus Biological cat#NBP2-67605), and CD35 (Novus Biological cat#NBP2-29460). Secondary antibodies include Discovery Red 610 (Roche Tissue Diagnostics cat#760–245), Discovery FITC (Roche Tissue Diagnostics cat#760–232), Discovery OmniMap anti-Rabbit HRP (Roche Tissue Diagnostics cat# 760–4311), and Discovery OmniMap anti-mouse HRP (Roche Tissue Diagnostics cat# 760–4310). Slides were mounted using ProLong Diamond Antifade mountant w/ DAPI (Invitrogen cat# P36971).

To obtain the low magnification views of the mesenteric lymph node, samples were imaged using a 10x/0.45 objective on a Zeiss digital slide scanning microscope (Axio Scan.Z1) equipped with a Colibri 7 LED illumination source and driven by ZEN Blue v. 3.1 (Carl Zeiss Microscopy). The samples were then imaged using either a 20x/0.8 or 63x/1.4 objective on a Zeiss laser scanning confocal microscope (LSM 880), driven by ZEN Black v. 2.3 (Carl Zeiss Microscopy). For the datasets acquired with the 63X/1.4 objective, image stacks were exported from ZEN software and deconvolved with Huygens Professional v. 20.10 (Scientific Volume Imaging) using the CMLE algorithm, with SNR = 20 and a maximum of 40 iterations.

### Generation of TRIM5 stable cell lines

PTM TRIMCyp was amplified from *Macaca nemestrina* cDNA derived from PBMCs. The pigtailed macaque TRIMCyp sequence was most similar to DQ308405 with the following amino acid changes: K44E, E209K, T269A, H373R, S438G. TRIMCyp was then cloned into pWPI DEST lentivirus vectors with an C-terminal HA tag. Rhesus TRIM5 with a C-terminal Flag tag, or an mCherry control gene, was cloned into pLenti6 by restriction enzyme cloning. Stable cell lines were generated by transfecting 5e6 293T cells with 1 μg pMD.G, 8 μg pSPAX2, and 8 μg pWPI vector using the ProFection Mammalian Transfection System (Promega). Supernatant was passed through a 0.45 micron filter and added to HEK293 cells. TRIM5 positive cells were selected with blasticidin S HCl at 10 μg/ml. Following construction of the stable TRIM5 cells, expression was validated by western blotting with an HA antibody (Cell Signaling Technology, mAb #2367) or a flag antibody (Cell Signaling Technology, mAb #14793).

### NGS library preparation

RNA was extracted from EDTA blood using TRIzol LS Reagent (ThermoFisher) according to the manufacturer’s protocol. Resulting RNA (140 μl) was extracted a second time using the QIAamp Viral RNA Mini kit (Qiagen). RNA was irradiated (10 megarad) for removal from the BSL4 according to established sample removal protocols [60]. RNA quality was analyzed using Agilent 2100 Bioanalyzer (Agilent Technologies), and 100 ng of RNA was treated with Qiagen FastSelect -H/M/R and -Globin kits to inhibit cDNA synthesis of rRNA and globin transcripts. cDNA synthesis and sequencing libraries were prepared following the Illumina Stranded RNA Prep, Ligation kit, with an additional 8 amplification cycles using the Kapa HiFi Hot Start amplification kit and assessed for library quality on BioAnalyzer High Sensitivity chips. The samples were quantified using the Kapa SYBR FAST Universal qPCR kit for Illumina sequencing (Kapa Biosystems, Boston, MA) on the CFX384 Real-Time PCR Detection System (Bio-Rad Laboratories, Inc, Hercules, CA). The libraries were normalized to 4 nM, pooled, denatured and further diluted to a 1.5 pM stock for clustering and paired-end 2 x 74 cycle sequencing on the Illumina NextSeq with one Mid Output and 2 High Output chemistry kits. Raw reads were trimmed of adapter sequence using cutadapt (https://cutadapt.readthedocs.io/en/stable/). The remaining reads were then filtered for low quality bases and reads using the FASTX-Toolkit (http://hannonlab.cshl.edu/fastx_toolkit/). Remaining reads were mapped to the NC_041754.1 *Macaca mulatta* genome, using HISAT2, and where sample appropriate, mapped to JF416958.1 Kyasanur forest disease virus strain P9605 or JF416954.1 Alkhumra hemorrhagic fever virus strain 200300001 using Bowtie2. Reads mapping to genes were counted using htseq-count [61]. Differential expression analysis was performed using the Bioconductor package DESeq2 [62].

### Functional analysis of transcriptomic data

Differential expression data was uploaded to Ingenuity Pathway Analysis (IPA; QIAGEN). IPA Core Analysis was run on differentially expressed genes were those defined as having a fold change > |1.5| relative to the 0 controls and *P* < 0.05. Enriched pathway lists were filtered by the Benjamini-Hochberg-corrected *P* value calculated by Fisher’s exact test (< 0.05) and the z-score (> |2|). Enriched pathways were also filtered based on annotation in the IPA knowledgebase. Within-pathway activation was predicted using the IPA Molecular Activity Prediction tool.

### Statistics

Statistics were performed as indicated with a two-way ANOVA followed by Sidaks multiple comparison test or Dunnett’s multiple comparison test. Multiplicity-adjusted *P* values are reported, and statistical significance are indicated by *P < 0.05, **P < 0.005, ***P < 0.0005, ****P < 0.0001. Error bars represent the standard deviation. All statistical analysis were performed using Graphpad Prism.

## Supporting information

S1 Fig Pigtail TRIMCyp does not restrict KFDV replication. **A.** PTM TRIMCyp-HA and rhesus macaque TRIM5-flag expression in HEK293 cells. **B.** HEK293 cells were infected with KFDV (MOI = 0.01), and supernatants were collected at T = 0, 24, and 48 hpi and titered on vero cells by limiting dilution plaque assays. Error bars indicate SD across replicate measurements. Statistics were performed on log-transformed data using Tukey’s multiple comparison test (*N* = 6; *P < 0.05, **P < 0.005, ***P < 0.0005, ****P < 0.0001).
(TIF)

S2 Fig Pigtailed macaques infected with KFDV by the sc route. **A.** Two pigtailed macaques were infected with 10^5^ pfu KFDV (sc). Clinical exams occurred on D0, 2, 4, 6, 8, 10, 14, 21, 28, 35, and 43 post infection. Animals were euthanized at day 43 post infection. **B.** Clinical scores for KFDV-infected animals. **C.** Quantitative real-time PCR detection of KFDV transcripts derived from plasma on D0 through D8 post infection. **D-E.** Dilutions of heat-inactivated sera were tested for neutralization of KFDV by plaque assay. Data were normalized to the D0 values, and error bars represent the SD across duplicate measurements.
(TIF)

S3 Fig KFDV positivity in the lung and brain. Tissues from KFDV sc/iv infected animals were examined for immunoreactivity with a KFDV RNAscope probe for viral RNA. Immunoreactivity was identified in **A.** bronchus-associated lymphoid tissue (100X, bar = 20 μm), **B.** choroid plexus (100X, bar = 20 μm), **C.** bronchus-associated lymphoid tissue (200X, bar = 50 μm), and **D.** choroid plexus (200X, bar = 50 μm).
(TIF)

S4 Fig Mesenteric lymph node double staining with AHFV and CD3, CD20, CD21, CD35, or CD68. Mesenteric lymph node sections from an animal infected sc/iv with AHFV was subjected to RNAscope and IHC for CD3, CD20, CD68, CD21, and CD35 using the RNAscope VS Universal ISH-IHC HRP fluorescent assay. AHFV (red) and cell markers (green). Scale bar for low-magnification CD3/AHFV panel, 500 μm; for high-magnification images of CD3, CD20, CD68, CD21, and CD35, 20 μm.
(TIF)

S5 Fig Mediastinal lymph node double staining with KFDV and DC68. Mediastinal lymph node sections from an animal infected sc/iv with KFDV was subjected to RNAscope and IHC for CD68. KFDV (red) and CD68 (green).
(TIF)

S1 Table Clinical observations of KFDV-infected pigtailed macaques by the subcutaneous route. (DOCX)
