# Supplementary figures and images for: A pigtailed macaque model of Kyasanur Forest disease virus and Alkhurma hemorrhagic disease virus pathogenesis
Source: PLoS Pathog. 2021 Dec 2;17(12):e1009678. doi: 10.1371/journal.ppat.1009678 (PMC8638978; doi:10.1371/journal.ppat.1009678)

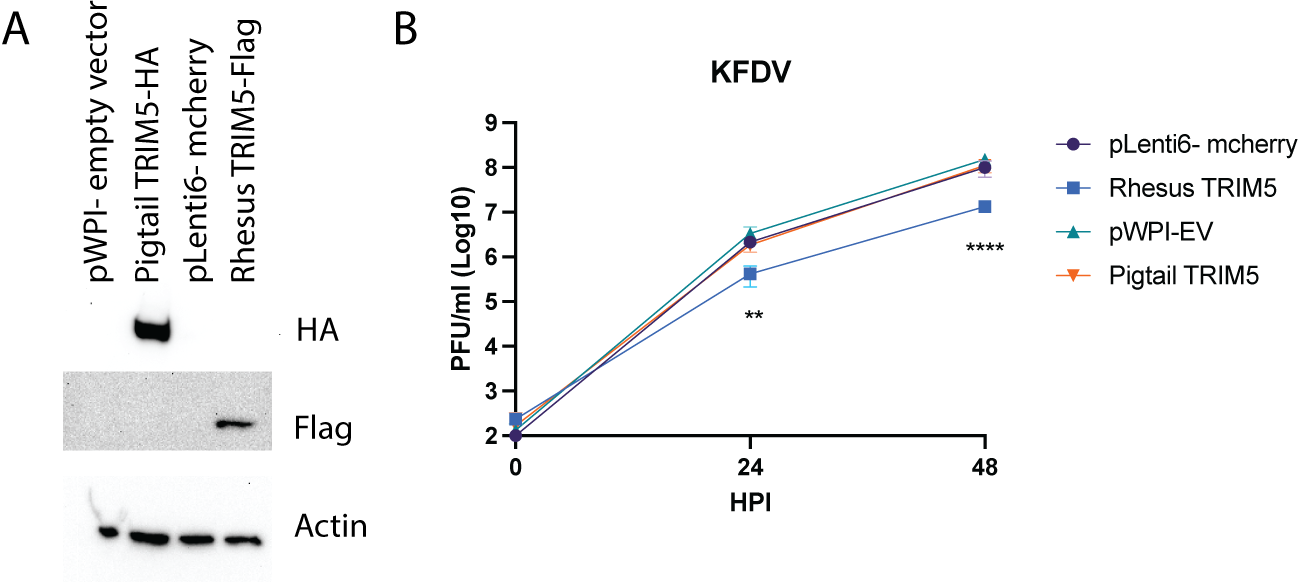

Supplement: S1 Fig — A. PTM TRIMCyp-HA and rhesus macaque TRIM5-flag expression in HEK293 cells. B. HEK293 cells were infected with KFDV (MOI = 0.01), and supernatants were collected at T = 0, 24, and 48 hpi and titered on vero cells by limiting dilution plaque assays. Error bars indicate SD across replicate measurements. Statistics were performed on log-transformed data using Tukey’s multiple comparison test (N = 6; *P < 0.05, **P < 0.005, ***P < 0.0005, ****P < 0.0001). (TIF) [file ppat.1009678.s001.tif]

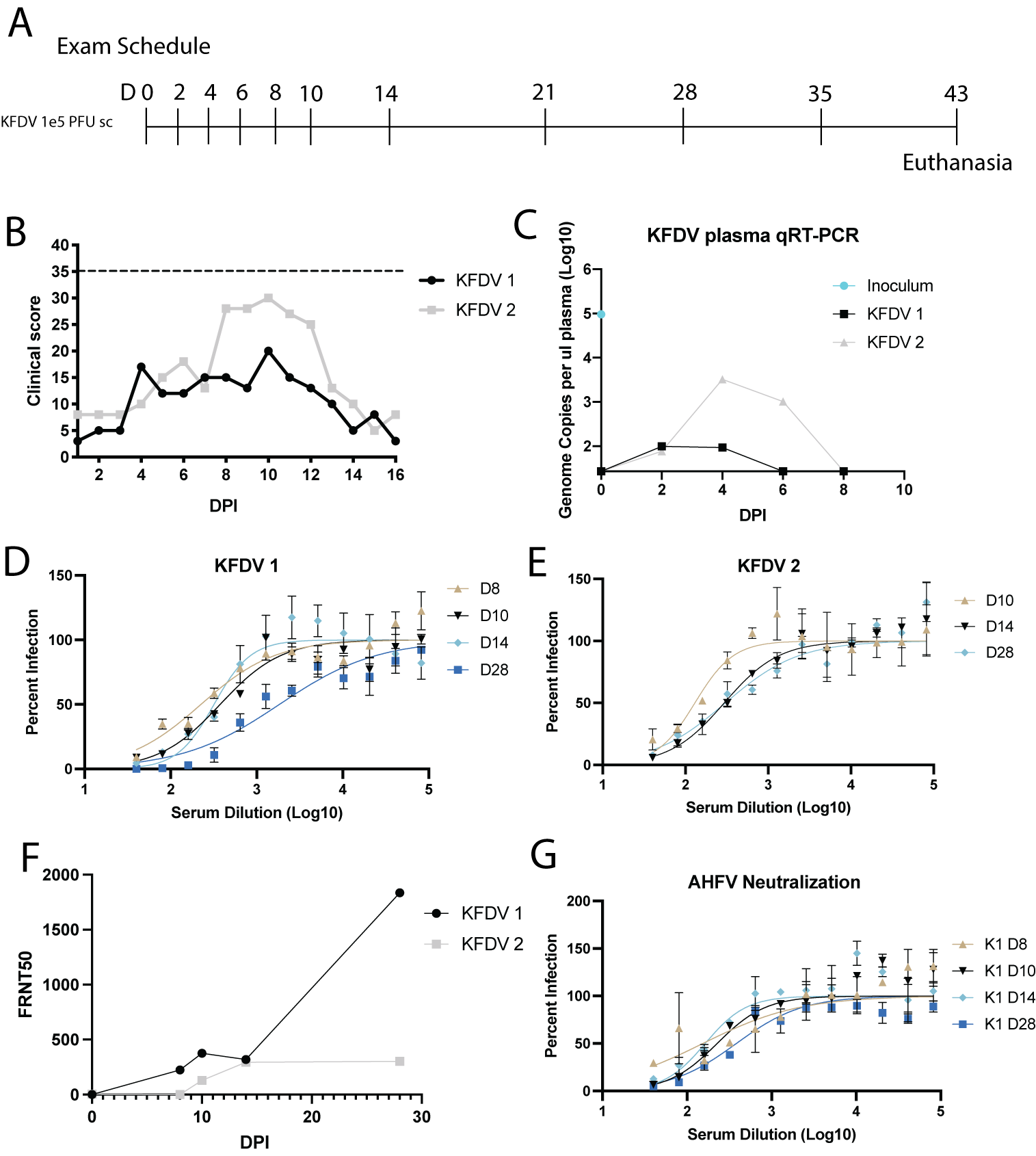

Supplement: S2 Fig — A. Two pigtailed macaques were infected with 105 pfu KFDV (sc). Clinical exams occurred on D0, 2, 4, 6, 8, 10, 14, 21, 28, 35, and 43 post infection. Animals were euthanized at day 43 post infection. B. Clinical scores for KFDV-infected animals. C. Quantitative real-time PCR detection of KFDV transcripts derived from plasma on D0 through D8 post infection. D-E. Dilutions of heat-inactivated sera were tested for neutralization of KFDV by plaque assay. Data were normalized to the D0 values, and error bars represent the SD across duplicate measurements. (TIF) [file ppat.1009678.s002.tif]

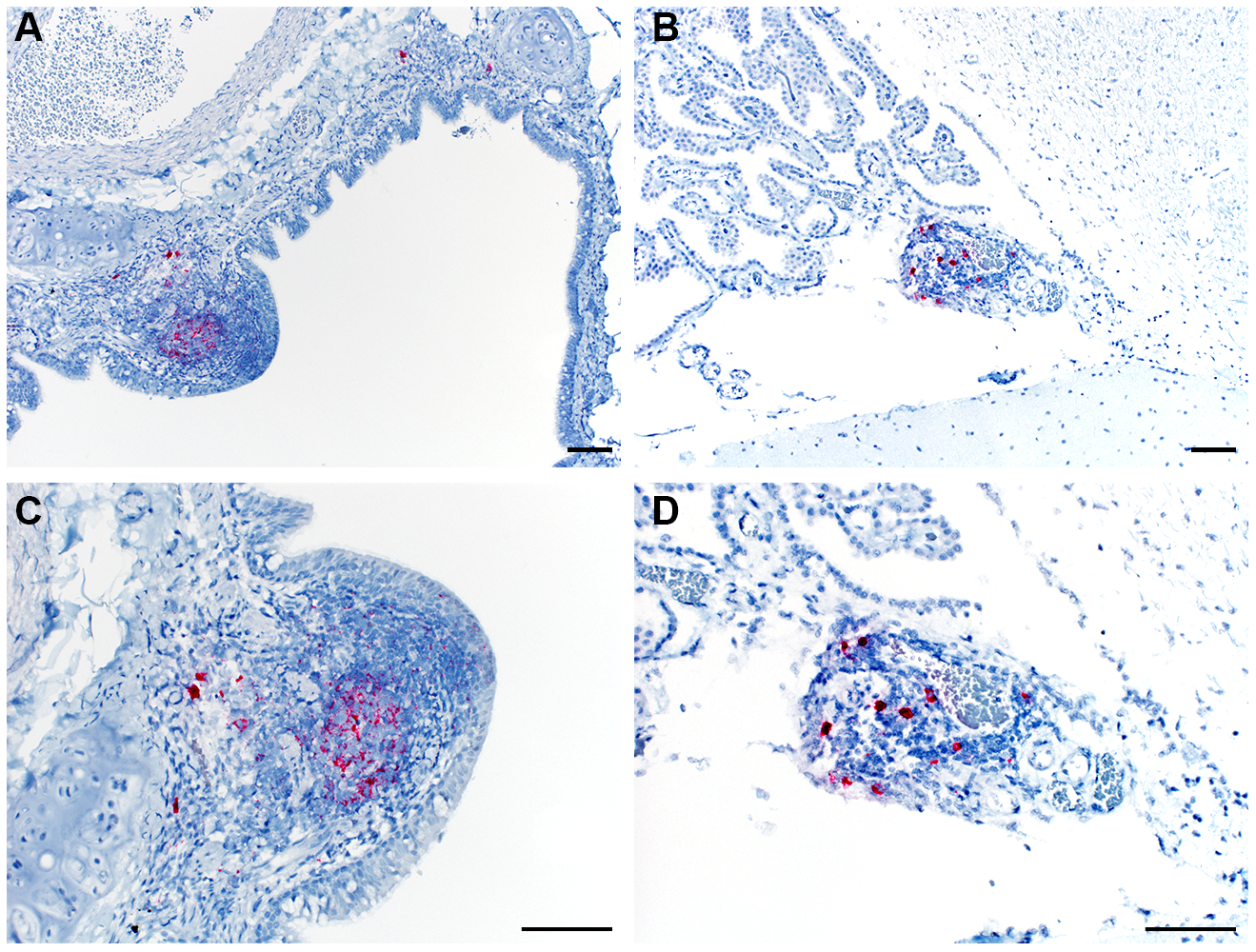

Supplement: S3 Fig — Tissues from KFDV sc/iv infected animals were examined for immunoreactivity with a KFDV RNAscope probe for viral RNA. Immunoreactivity was identified in A. bronchus-associated lymphoid tissue (100X, bar = 20 μm), B. choroid plexus (100X, bar = 20 μm), C. bronchus-associated lymphoid tissue (200X, bar = 50 μm), and D. choroid plexus (200X, bar = 50 μm). (TIF) [file ppat.1009678.s003.tif]

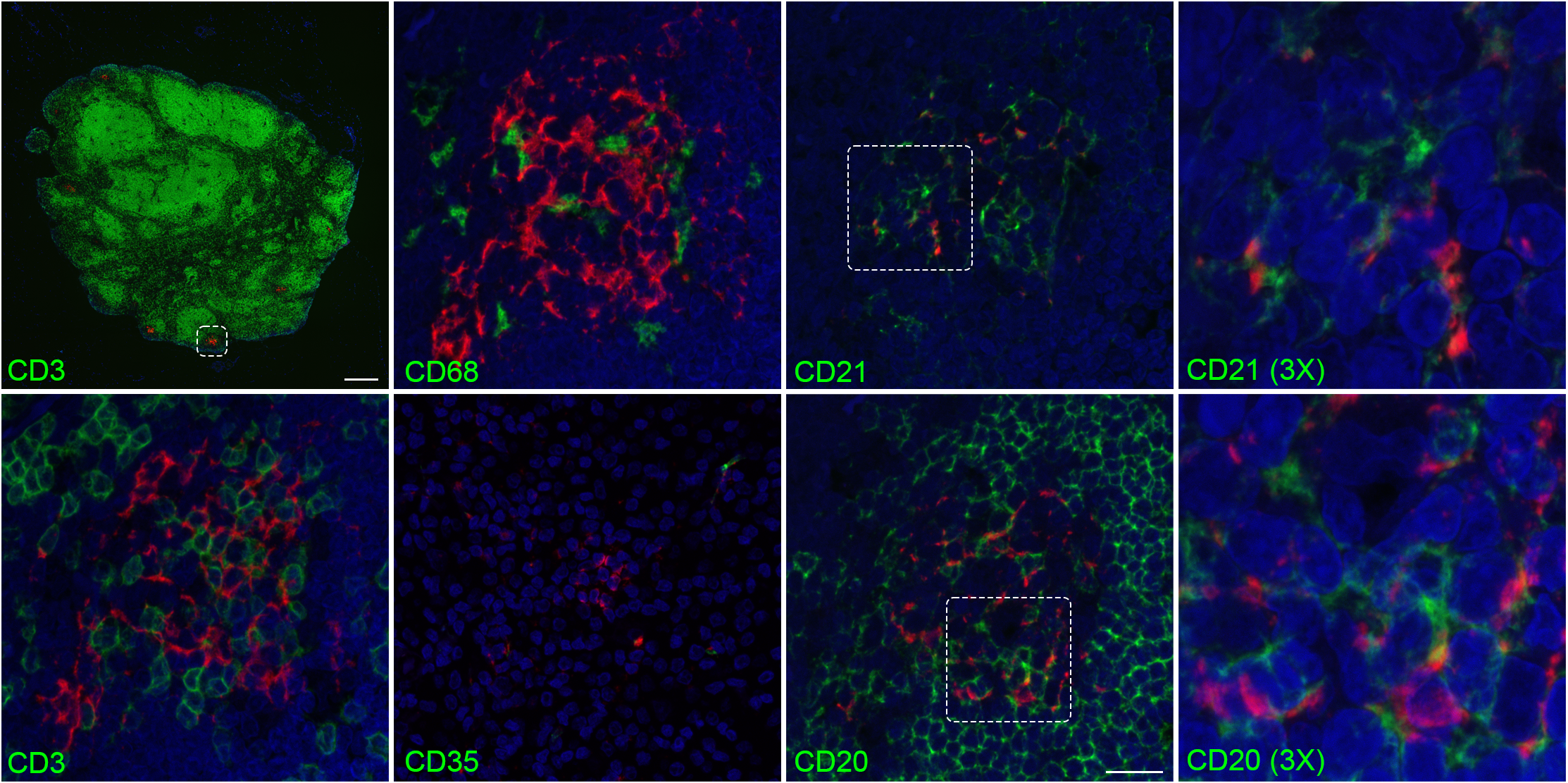

Supplement: S4 Fig — Mesenteric lymph node sections from an animal infected sc/iv with AHFV was subjected to RNAscope and IHC for CD3, CD20, CD68, CD21, and CD35 using the RNAscope VS Universal ISH-IHC HRP fluorescent assay. AHFV (red) and cell markers (green). Scale bar for low-magnification CD3/AHFV panel, 500 μm; for high-magnification images of CD3, CD20, CD68, CD21, and CD35, 20 μm. (TIF) [file ppat.1009678.s004.tif]

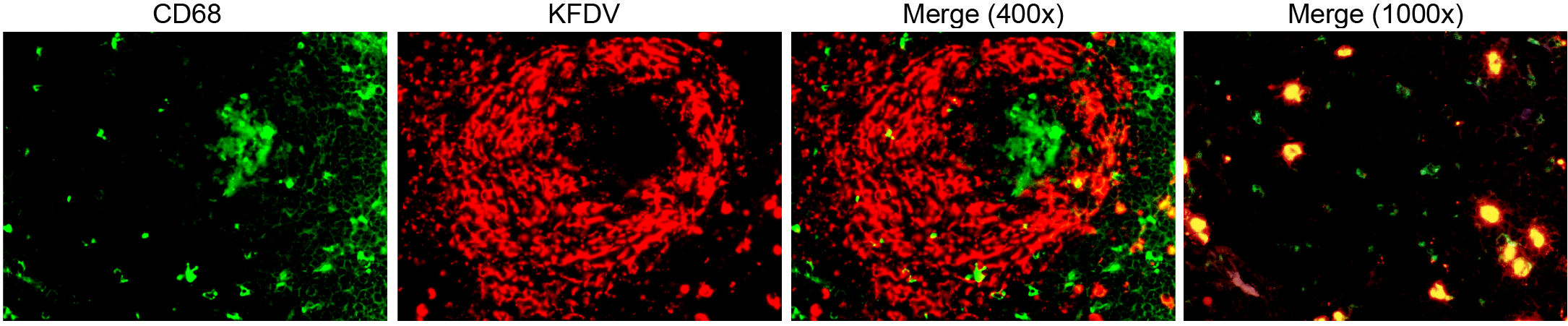

Supplement: S5 Fig — Mediastinal lymph node sections from an animal infected sc/iv with KFDV was subjected to RNAscope and IHC for CD68. KFDV (red) and CD68 (green). (TIF) [file ppat.1009678.s005.tif]
