# Supplementary material for: A pigtailed macaque model of Kyasanur Forest disease virus and Alkhurma hemorrhagic disease virus pathogenesis
Source: PLoS Pathog. 2021 Dec 2;17(12):e1009678. doi: 10.1371/journal.ppat.1009678 (PMC8638978; doi:10.1371/journal.ppat.1009678)
Supplement: S1 Table — (DOCX) [file ppat.1009678.s006.docx]

|  | KFDV 1 (sc) | KFDV 2 (sc) | Frequency (sc) n = 2 |
| --- | --- | --- | --- |
| Decreased appetite | Severe | Severe | 100% |
| Loss of interest in treats | N | N | 0% |
| Piloerection | Y | N | 50% |
| Hunched posture | Y | N | 50% |
| Loose stool | Y | N | 50% |
| No feces | N | N | 0% |
| clear nasal discharge | Y | Y | 100% |
| Transient epistaxis | N | Y | 50% |
| Unsteady | N | N | 0% |
| Slow and careful movements | N | N | 0% |
| Reluctant to move | N | N | 0% |
| Using cage for support | N | N | 0% |
| Dehydration | N | N | 0% |
| CRT 2 seconds or more | D10, 3 sec. | N | 50% |
| Temperature change > 1 °C | N | N | 0% |
| Flushed Appearance | N | N | 0% |
| Mild Facial edema | N | N | 0% |
| Other Comments |  |  |  |
| Score 35 | N | N | 0% |

**S1 Table. Clinical observations of KFDV-infected pigtailed macaques by the subcutaneous route.**
